# Association Between Medicaid Managed Care Coverage of Substance Use Services and Treatment Utilization

**DOI:** 10.1001/jamahealthforum.2022.2812

**Published:** 2022-08-26

**Authors:** Samantha G. Auty, Megan B. Cole, Jacob Wallace

**Affiliations:** 1 Department of Health Law, Policy and Management, Boston University School of Public Health, Boston, Massachusetts; 2 Department of Health Policy and Management, Yale School of Public Health, New Haven, Connecticut

## Abstract

**Importance:**

Medicaid insures a disproportionate share of adults with substance use disorder (SUD) and is thus uniquely positioned to facilitate access to care. Many enrollees receive coverage through Medicaid managed care (MMC) plans, which receive capitated payments in exchange for coverage of a defined set of benefits. Historically, coverage of substance use services has been carved out of MMC plans and financed fee-for-service (FFS) by state Medicaid programs, but in recent years, many states have opted to carve in this benefit. Little is known about whether MMC coverage of substance use services, relative to FFS coverage, is associated with changes in utilization.

**Objective:**

To examine the association between changes in MMC coverage of substance use services and admissions for substance use treatment.

**Design, Setting, and Participants:**

This cross-sectional study examined changes in admissions for substance use treatment in 2 states after coverage of substance use services was either carved into (Nebraska) or carved out of (Maryland) comprehensive MMC coverage. Synthetic control methods were used to compare changes in admissions between states that did and did not alter MMC coverage of substance use services. Data on substance use treatment admissions were obtained from the Treatment Episode Data Set—Admissions from 2010 to 2019.

**Exposures:**

Carve-outs or carve-ins of coverage for both inpatient and outpatient substance use services from comprehensive MMC coverage.

**Main Outcomes and Measures:**

Reported substance use treatment admissions per 100 000 residents and admissions by treatment type (ie, rehabilitation or residential, outpatient, and detoxification) per 100 000 residents.

**Results:**

Maryland’s carve-out was associated with an additional mean 787.1 (95% CI, 624.6-1141.7) substance use admissions per 100 000 residents during 2015 and 2016, a relative increase of 104.4% (95% CI, 64.4%-154.1%) compared with its synthetic control. This increase was concentrated among changes in outpatient services utilization. In Nebraska, the carve-in was associated with a mean decrease of 97.2 (95% CI, −23.4 to 213.6) admissions per 100 000 residents, a relative decrease of 33.2% (95% CI, −54.1% to 29.6%) compared with its synthetic control and was concentrated primarily among admissions for detoxification services.

**Conclusions and Relevance:**

The results of this cross-sectional study suggest that carving out coverage of substance use services and financing them through FFS coverage may be associated with overall increases in treatment utilization but with heterogeneous associations across states and treatment types.

## Key Points

**Question:**

Did admissions for substance use treatment change after coverage of substance use services was carved into or carved out of comprehensive Medicaid managed care in 2 states?

**Findings:**

In this cross-sectional study of 2010 to 2019 data from the Treatment Episode Data Set—Admissions, Maryland experienced a 104.4% relative increase in admissions following the carve-out and Nebraska experienced a 33.2% relative decrease following the carve-in compared with each state’s respective synthetic control.

**Meaning:**

These results suggest that carving out coverage of substance use services from Medicaid managed care may be associated with increased treatment utilization, but changes in treatment utilization may be heterogeneous across states.

## Teaser

This cross-sectional study of data from the Treatment Episode Data Set—Admissions examines the association between changes in Medicaid coverage of substance use services and admissions for substance use treatment in Maryland and Nebraska.

## Introduction

Improving access to substance use disorder (SUD) treatment has been a long-standing priority in the US, but there are still significant gaps between need and access. In 2020, an estimated 20.3 million people had an SUD, but only 2.6 million received any treatment.^1^ Given that a disproportionate share of persons with SUDs are enrolled in Medicaid,^2^ states are uniquely positioned to improve access to substance use treatment services.

Over the past several decades, most states transitioned from fee-for-service (FFS) Medicaid, wherein states design coverage and pay practitioners directly, to Medicaid managed care (MMC). In MMC, benefits are delivered by private health plans, which receive capitated payments from state Medicaid programs in exchange for coverage of a defined set of services.^3^ This model creates strong incentives for MMC plans to control cost. Ideally, this involves steering enrollees toward high-value services and practitioners and limiting the use of low-value services. However, plans could also respond to capitation incentives by stinting on needed care, despite regulatory requirements to monitor and report on quality of care.

Historically, coverage of behavioral health services—including substance use treatment—has presented a challenge in MMC. One purported benefit of MMC is care coordination among networks of high-quality practitioners, but the limited supply of behavioral health practitioners has hampered MMC plans’ efforts to construct behavioral health networks.^4^ In addition, behavioral health services can be costly, and MMC plans may not be able to recoup these costs through capitated payments, even when payments are risk adjusted to account for differences in case mix.^5^ Given these challenges, states historically “carved out” behavioral health coverage, paying for it through either FFS Medicaid or separate behavioral care organizations, which may or may not face financial risk for the coverage of services.^4^ One study of these carve-outs found inconsistent associations with utilization of substance use services.^4^ However, there is limited evidence regarding the associations of MMC coverage of substance use services—and the higher-powered incentives they create for cost control—with utilization since the implementation of the Affordable Care Act.

Medicaid managed care carve-outs of behavioral health coverage may expand or restrict access to substance use services. For example, carve-outs could increase care fragmentation, in which the separate financing of physical and behavioral health may make it more difficult for enrollees to navigate their provider networks and limit opportunities for care coordination by the health plan.^6^ Moreover, MMC plans are subject to more stringent regulations relating to network adequacy and plan quality than Medicaid FFS.^7^ On the other hand, carve-outs could improve access to SUD services if provider networks are more expansive or if enrollees encounter fewer utilization-management tools (eg, prior authorization) in FFS models. As of 2021, only 5 states financed SUD services through FFS Medicaid.^7^ This includes Maryland, which carved out inpatient and outpatient SUD services from Medicaid in 2015, and Nebraska, which “carved in” these services to Medicaid in 2017. In this study, we examined the association of Maryland’s carve-out and Nebraska’s carve-in with substance use treatment admissions using synthetic control methods (SCM).

## Methods

Boston University’s Institutional Review Board (IRB) approved this study and waived the need for informed consent because only deidentified, publicly available data were used. This study followed the Strengthening the Reporting of Observational Studies in Epidemiology (STROBE) reporting guideline for cross-sectional studies.

### Data and Sample

This study used data from 2010 to 2019 on all admissions to substance use treatment facilities from the Treatment Episode Data Set—Admissions (TEDS-A), collected by the Substance Abuse and Mental Health Services Administration.^8^ Substance use treatment facilities that receive any public funds are required to report data on all admissions, including information on patient demographic characteristics, substance use characteristics, and treatment plan. The TEDS-A captures most admissions to substance use treatment facilities.^9^ Carve-out policies for inpatient and outpatient substance use services were identified using surveys from Kaiser Family Foundation,^10^ which maintains archived data from the Centers for Medicare & Medicaid Services, and confirmed via state filings.

Additional state-level data were also acquired. Data on MMC enrollment and Medicaid income eligibility for childless adults were obtained from the Kaiser Family Foundation.^11^ Data on state use of MMC “in lieu of” authority, which allows MMC plans to cover services delivered at institutes of mental disease (facilities with more than 16 beds that provide care primarily for persons with mental illness or SUD) and receive federal matching funds for those enrollees, were also obtained from Kaiser Family Foundation. State-level data on drug overdose deaths and population were drawn from the Centers for Disease Control and Prevention WONDER (Wide-ranging Online Data for Epidemiologic Research) database.^12^ Drug overdose deaths were identified using *International Statistical Classification of Diseases and Related Health Problems, Tenth Revision* (*ICD-10*) codes X40 to 44, X60 to 64, X85, and Y10 to 14. Data on the supply of substance use facilities at the state level were obtained from the National Survey of Substance Abuse Treatment Centers collected by the Substance Abuse and Mental Health Services Administration.

States were included in the analysis if they had no missing years of TEDS-A data, used any comprehensive MMC during the study period, and did not change between FFS and MMC after 2014. Four states (South Carolina, Oregon, Washington, and Georgia) were identified by the Substance Abuse and Mental Health Services Administration as having incomplete TEDS-A data for several years during the study period and were excluded. Fourteen states (Alabama, Alaska, Arkansas, Colorado, Connecticut, Idaho, Maine, Montana, North Carolina, North Dakota, Oklahoma, South Dakota, Vermont, and Wyoming) were excluded because they did not use comprehensive MMC during the study period. Iowa transitioned to MMC during 2015 and 2016 and was excluded. Arizona fully integrated behavioral and physical health services under comprehensive MMC coverage for all enrollees in 2018 and was excluded. Our final analytic data set included 310 state-years from 30 states and the District of Columbia.

### Primary Exposure

The primary exposure was carving out or carving in of both inpatient and outpatient SUD services. Maryland carved out all substance use services starting on January 1, 2015, and financed them FFS through an administrative service organization within the state Medicaid program.^13^ Prior to 2015, these services were carved in. Nebraska carved in inpatient and outpatient substance use services to risk-based comprehensive MMC coverage starting on January 1, 2017.^14^ Prior to Nebraska’s carve-in, these services were managed through a separate behavioral health organization.

### Primary Outcome

The primary outcome was utilization of substance use treatment, measured as total annual substance use treatment admissions per 100 000 residents; this included admissions for inpatient rehabilitation or residential services, ambulatory outpatient (intensive and nonintensive) services, and detoxification (detox) services. Secondary outcomes included admissions by treatment type, including rehabilitation or residential, ambulatory outpatient, and detox, per 100 000 residents.

### Covariates

Rates of drug overdose deaths were calculated as the number of deaths per 100 000 residents by state-year. Medicaid managed care penetration was the proportion of Medicaid enrollees covered by comprehensive MMC plans by state-year. The supply of substance use treatment facilities and the supply of these facilities receiving public funds was expressed as the number of each facility type per 100 000 residents. States were considered as having expanded Medicaid if they extended Medicaid eligibility to childless adults earning 138% of the federal poverty limit as of the study year.

### Statistical Analysis

#### Synthetic Control Methods

Synthetic control methods were used to estimate the association between comprehensive MMC coverage of substance use services (ie, carved in vs carved out benefit) and utilization of these services; SCMs create a synthetic version of a treated unit (eg, state or county) using data from nontreated units in a donor pool. The synthetic control is a weighted average of units in the donor pool, with weights chosen by a data-driven algorithm so that pretrends in covariates and/or outcomes are as similar as possible between the treated unit and synthetic control.^15,16^

We first identified unadjusted changes in all substance use treatment admissions and admissions stratified by treatment type by comparing admissions rates before the carve-out (Maryland preperiod: 2010-2014) or carve-in (Nebraska preperiod: 2012-2016) with the subsequent 2 years in each state. This 2-year postperiod was selected to avoid other potentially confounding policy changes in these states (ie, Maryland implemented a 1115 demonstration waiver to improve SUD treatment in July 2017).

We then used SCMs to create weighted combinations of control states (ie, synthetic Maryland and Nebraska) using established approaches.^3,17^ Weights were generated so that synthetic controls were similar to treated states on pretrends of opioid deaths per capita, MMC penetration, Medicaid expansion status, substance use facilities per capita, earmarked substance use facilities per capita, and substance use treatment admissions. We matched on substance use treatment admissions in only the first year of the preperiod and all other covariates over the entire preperiod (see eTables 1 and 2 in the Supplement for state weights in each synthetic control model). Matching on outcomes for the entire preperiod can inflate effect estimates,^18^ and lagging outcome data mitigates potential bias.

Separate synthetic control models were estimated for each treated state. Taylor series linearization was used to calculate 95% confidence intervals. As is standard in the literature,^15^ we conducted permutation tests to determine placebo effect sizes by iteratively reassigning treatment status to each state in the donor pool and rerunning synthetic control models, which generates placebo effect sizes. Placebo effect sizes generated from permutation tests do not always comport with confidence intervals obtained from Taylor series linearization, particularly with a small number of treated units. To estimate synthetic controls, we used the R software microsynth package, version 4.1.3 (R Foundation for Statistical Computing).^17^

#### Robustness Tests

Several robustness tests were conducted. The MMC carve-outs likely do not affect persons with non-Medicaid insurance coverage, and thus we would expect observed changes in utilization of substance use services to be associated with services financed by Medicaid. While the TEDS-A data include information on source of health insurance coverage, this variable is often missing in a number of states. To assess whether the results of carve-outs were concentrated among changes in admissions among Medicaid enrollees, we reran synthetic control models stratified by source of health insurance coverage using states with a low degree of missingness in this variable (see the eAppendix in the Supplement for additional details). We also reestimated synthetic control models with a more expansive donor pool of states by including those that use FFS Medicaid. Finally, because a best practice for variance estimation in SCMs has not yet been established, we estimated prediction intervals using the approach developed by Cattaneo et al in their empirical example, wherein they vary the approach to estimate prediction intervals to quantify in- and out-of-sample uncertainty.^19^ To obtain prediction intervals for synthetic control models, we used the R software scpi package (R Foundation for Statistical Computing). We then used a 2-sided *t* test to determine whether the observed effect in treated states exceeded the placebo effects in control states at the α = .05 significance level.

## Results

### Maryland

In the 2 years following the carve-out of substance use services from MMC coverage in Maryland, treatment admissions increased by a mean of 838.3 (95% CI, 414.3-1262.3) per 100 000 residents. Following the carve-out, admissions to ambulatory outpatient treatment increased by a mean of 969.4 (95% CI, 652.9 to 1285.9) per 100 000 residents, while admissions per 100 000 residents for rehabilitation or residential services (−101.9 [95% CI, −220.0 to 16.3] per 100 000 residents) and detox (−29.3 [95% CI, −56.9 to −1.6] per 100 000 residents) decreased (Table).

**Table.** Changes in Per Capita Admissions for Substance Use Treatment Associated With Changes in MMC Coverage

Type of admission	Unadjusted admissions per 100 000 state residents, mean (SD)^a^		Synthetic control estimates, % change (95% CI)^b^
	Before policy change	After policy change	
**Carve-out: Maryland**			
All admissions	897.9 (193.8)	1736.3 (209.9)	104.4 (64.4 to 154.1)
Outpatient admissions	642.3 (129.3)	1611.7 (203.4)	170.6 (116.9 to 237.6)
Inpatient rehabilitation or residential admissions	162.4 (61.4)	60.6 (12.7)	−42.3 (−59.5 to −17.9)
Detox admissions	93.2 (13.1)	63.9 (8.2)	−35.3 (−66.7 to 3.3)
**Carve-in: Nebraska**			
All admissions	751.1 (83.2)	662.5 (63.5)	−33.2 (−54.1 to −29.6)
Outpatient admissions	289.9 (35.7)	405.9 (10.1)	26.9 (−28.7 to 89.0)
Inpatient rehabilitation or residential admissions	95.6 (9.9)	104.4 (2.9)	6.5 (−20.3 to 42.3)
Detox admissions	320.2 (13.5)	192.2 (84.5)	−38.4 (−61.3 to −1.9)

Abbreviations: detox, detoxification treatment; MMC, Medicaid managed care.

^a^ Mean (SD) admissions during the 5 years immediately preceding and 2 years following changes in MMC coverage of substance use services.

^b^ Synthetic control estimates for the percentage change in outcomes during the 2 years after coverage change.

**Figure 1.**
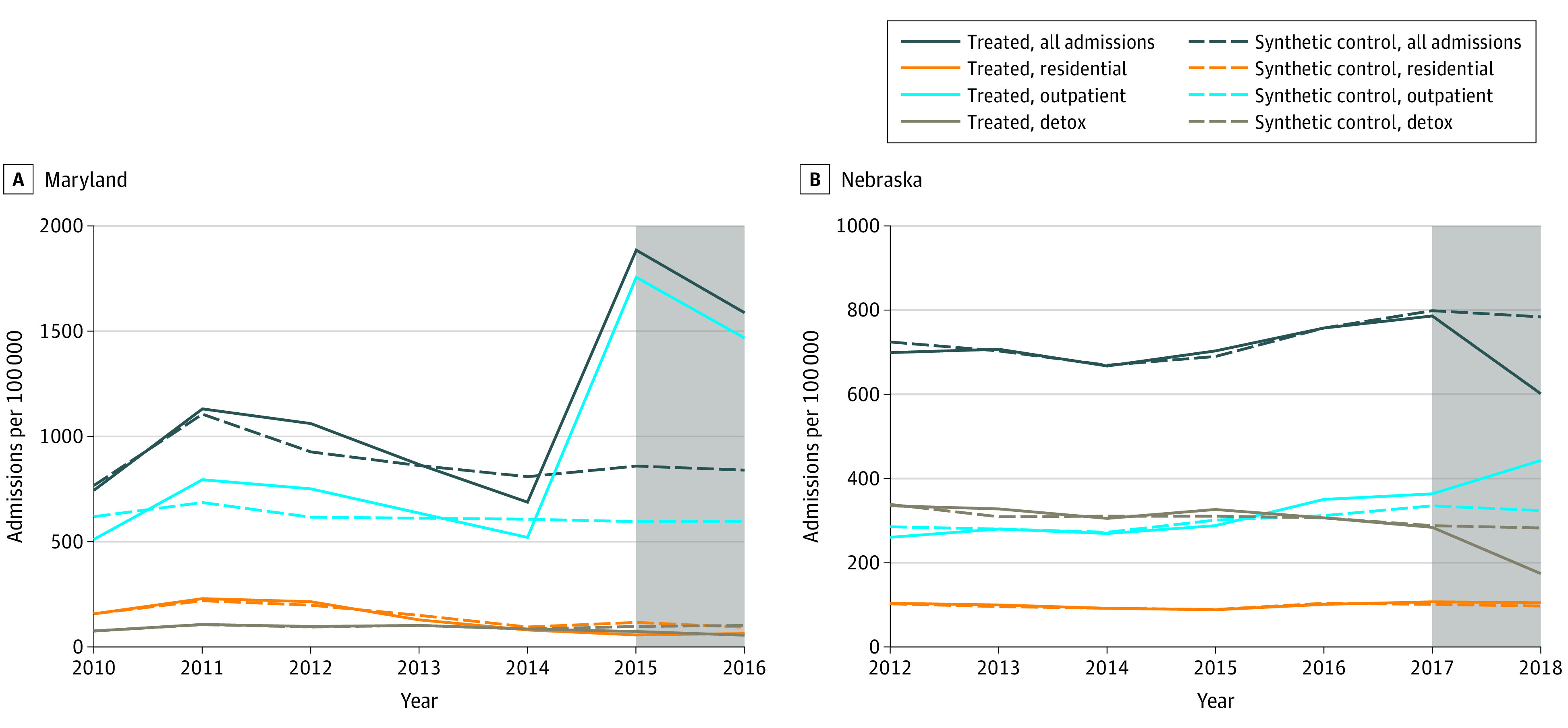
Trends in Substance Use Treatment Admissions Overall and by Type of Service per 100 000 Residents in Study vs Synthetic Control States Maryland carved out all substance abuse disorder services from Medicaid managed care (MMC) in 2015, and Nebraska carved in these services to comprehensive MMC coverage in 2017. The shaded area of the graphs indicates each state’s postperiod. Detox indicates detoxification treatment. Synthetic control is a weighted average of units (ie, states) in the donor pool, with weights chosen by a data-driven algorithm so that pretrends in covariates and/or outcomes are as similar as possible between the treated unit and synthetic control.

**Figure 2.**
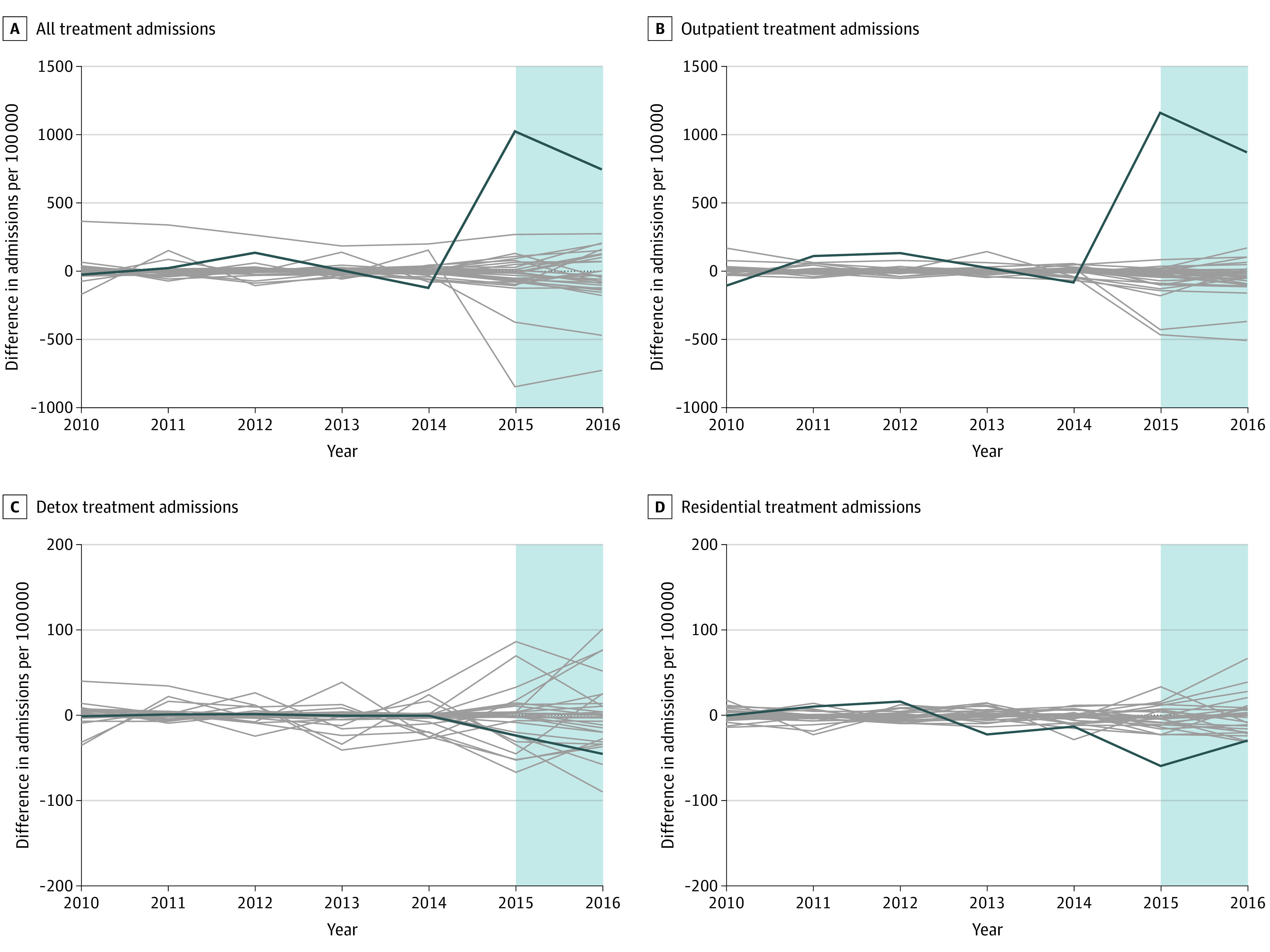
Permutation Tests of Admissions for Substance Use Services Overall and by Type of Service Provided in Maryland and Synthetic Controls Synthetic control is a weighted average of units (ie, states) in the donor pool, with weights chosen by a data-driven algorithm so that pretrends in covariates and/or outcomes are as similar as possible between the treated unit and synthetic control. The blue shaded area indicates the postperiod (2015 and 2016). Gray lines represent the estimated placebo differences in outcomes between individual control states and their respective synthetic controls.

Pretrends for synthetic controls closely mirrored those in Maryland (Figure 1A). In synthetic control models, the carve-out was associated with an additional mean 787.1 (95% CI, 624.6-1141.7) substance use admissions per 100 000 residents from 2015 and 2016, a relative increase of 104.4% (95% CI, 64.4%-154.1%) (Table). Similar findings were not observed in any permutation tests (0 of 30 tests) (Figure 2A). Synthetic control models stratified by treatment type found that the carve-out was associated with an additional mean 1015.2 (95% CI, 640.2-1277.2) admissions per 100 000 residents for ambulatory outpatient services compared with the synthetic control, a relative increase in admissions of 170.6% (95% CI, 116.9%-237.6%) per 100 000 residents. Similar findings were not observed in any permutation tests (0 of 30 tests) (Figure 2B). No significant changes in admissions for other types of services (ie, rehabilitation or residential or detox) were observed (Table).

### Nebraska

In the 2 years following the carve-in of substance use services to comprehensive MMC coverage in Nebraska, admissions for substance use services decreased by a mean of 90.6 (95% CI, −21.9 to 201.2) per 100 000 residents. Admissions for ambulatory outpatient services increased by a mean of 115.9 (95% CI, 62.8-168.9) per 100 000 residents, while detox admissions decreased (mean decrease, −127.9 [95% CI, −38.2 to −217.7] per 100 000 residents). Admissions to rehabilitation or residential facilities were largely unchanged (Table).

**Figure 3.**
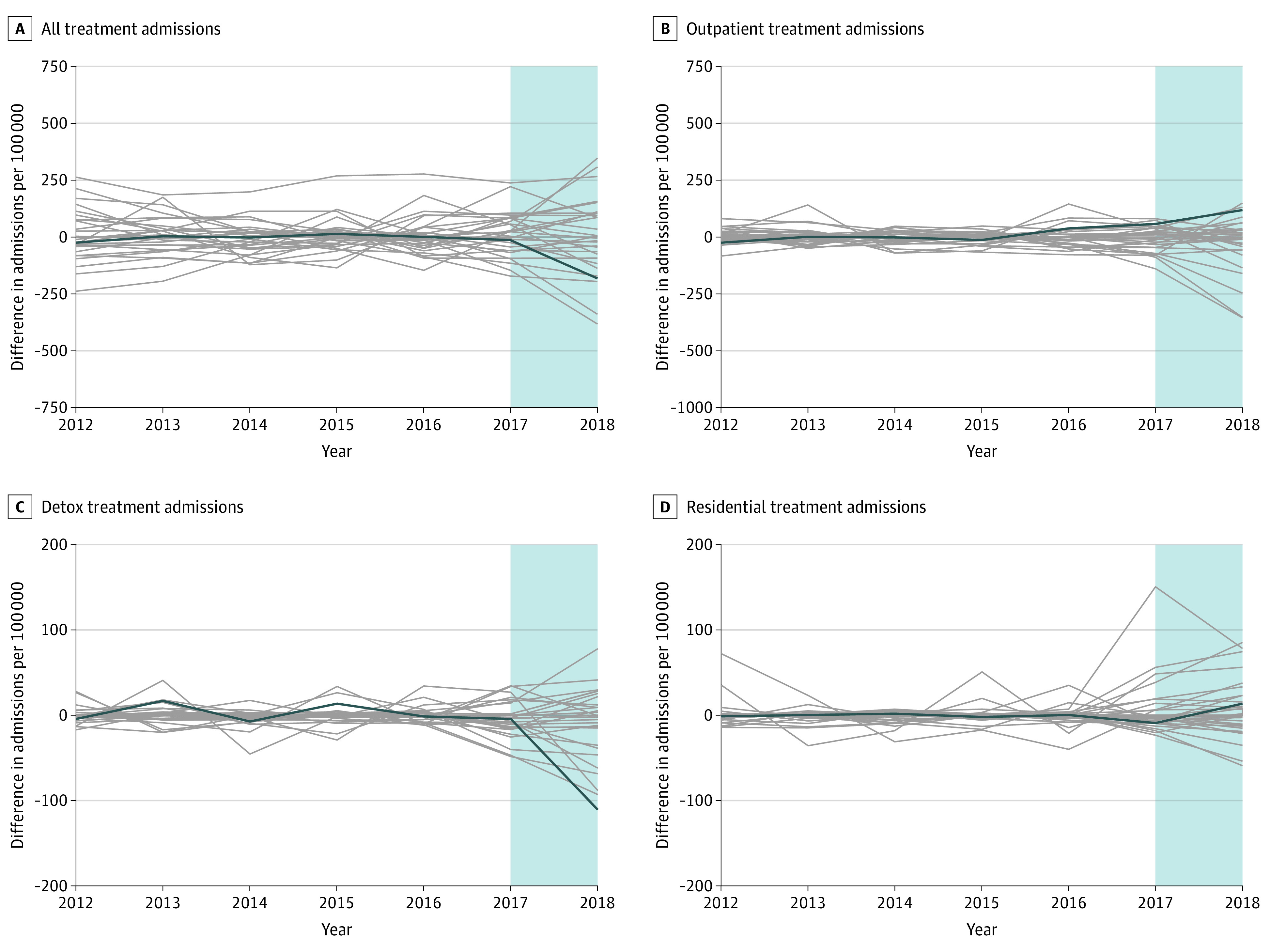
Permutation Tests of Admissions for Substance Use Services Overall and by Type of Service Provided in Nebraska and Synthetic Controls Synthetic control is a weighted average of units (ie, states) in the donor pool, with weights chosen by a data-driven algorithm so that pretrends in covariates and/or outcomes are as similar as possible between the treated unit and synthetic control. The blue shaded area indicates the postperiod (2017 and 2018). Gray lines represent the estimated placebo differences in outcomes between individual control states and their respective synthetic controls.

Pretrends for synthetic controls closely mirrored those in Nebraska prior to the carve-in of substance use services (Figure 1B). In synthetic control models, the carve-in was associated with a mean decrease of 97.2 (95% CI, −23.4 to 213.6) admissions per 100 000 residents, a relative decrease of −33.2% (95% CI, −54.1% to 29.6%) admissions per 100 000 residents (Table). However, similar findings were observed in several permutation tests (4 of 30 tests). Synthetic control models stratified by treatment type found that the carve-in was associated with a decrease of admissions for detox services compared with its synthetic control, a relative decrease in admissions per 100 000 residents of 38.4% (95% CI, −61.3% to −1.9%), although similar findings were not observed in any permutation tests (Figure 3). However, a nearly identical increase was observed in ambulatory outpatient admissions (mean, 26.9% [95% CI, −28.7% to 89.0%]) compared with its synthetic control (Table), but findings of similar magnitude were found in several permutation tests (2 of 30 tests) (Figure 3). No significant changes in admissions for rehabilitation or residential services were observed.

### Robustness Tests

Synthetic control models stratified by source of health insurance coverage found that changes in admissions in Maryland and Nebraska were concentrated among Medicaid-covered admissions in both states and that admissions did not change for non-Medicaid insurance coverage following MMC coverage changes. For both Nebraska and Maryland, synthetic control models rerun using an unrestricted donor pool of states found directionally consistent and similar estimates of changes in admissions following MMC coverage changes (eTable 3 in the Supplement). Similar results were also obtained using the methods developed by Cattaneo et al.^19^

## Discussion

This cross-sectional study found that carving out coverage of substance use services from MMC coverage was associated with increases in reported treatment admissions for substance use, while carving these services into MMC was associated with decreases in reported admissions. These results suggest that utilization of substance use treatment may be partially dependent on the Medicaid payment model and related policy changes.^20^ However, the exact mechanism is unclear. First of all, not all MMC plans are created equal within a state,^19^ but there is also significant variation in MMC program design between states.^18^ This is particularly true of Medicaid coverage for substance use services, as almost all states impose restrictions to access certain services.^21^ Moreover, states vary both in the extent of their investment in and oversight of their MMC programs, making it difficult to generalize from the experience of one state to another.

To constrain costs, MMC plans to have tools to steer beneficiaries to lower-cost settings (eg, short-term stays at an institute of mental disease rather than the costlier hospital inpatient setting) or higher-value therapies. For example, plans may use restrictive provider networks^22^ or utilization-management tools. The evidence^23^ suggests that these are both blunt tools for reducing health care spending, leading to reductions in the use of both necessary and unnecessary care, but little is known about this tradeoff for substance use services. Notably, we find that Nebraska experienced a large decrease in admissions for detoxification and a concomitant increase in ambulatory outpatient admissions after substance use benefits were carved in to comprehensive MMC coverage. This may represent a shift to higher quality, evidence-based care that improves the health of enrollees in the long run, but service-level data to confirm this are lacking. Nevertheless, this result suggests that enrollees were steered toward different services after substance use coverage was carved into MMC.

In Maryland, we found evidence of substantial increases in ambulatory outpatient admissions after coverage of substance use services was carved out of MMC and financed through FFS Medicaid. Preliminary evidence from a state-led evaluation did not find an increase in the number of unique Medicaid enrollees receiving substance use services after the carve-in,^13^ suggesting that our result may primarily be associated with increases in treatment admissions per enrollee rather than the unique number of enrollees in treatment. However, we are unable to test this hypothesis in our data. This finding may be associated with other policies Maryland implemented around the time of the carve-in, including relaxing utilization limits for SUD services. As a result of this complex bundle of policies, the marked increase in admissions for substance use services in Maryland may not be generalizable to carve-outs in other states. However, the results are directionally similar to Nebraska’s, providing a collage of evidence suggesting that Medicaid coverage policies shape utilization of SUD services.

### Limitations

This study has several limitations. First, it is possible that MMC coverage steers enrollees toward facilities less likely to report treatment admissions to TEDS-A or alters the composition of facilities that report treatment admissions. If true, our study may overestimate the effect of carving the coverage of substance use services out of MMC. While the majority of treatment facilities in the US are required to report admissions,^24^ we cannot rule out the possibility that sample selection bias contributes to our result. Second, the TEDS-A data contain all treatment admissions, not just those of patients with Medicaid coverage, but inclusion of admissions not covered through Medicaid would tend to bias estimates away from the null. Third, we are unable to account for all other changes in Medicaid policies that might affect access to substance use services, including changes in Medicaid policy coinciding with the carve-in or carve-out of substance use disorder services from MMC. Specifically, Maryland relaxed limits on utilization of SUD services, introducing the possibility that observed increases may reflect pent-up demand, rather than persistent increases in substance use services utilization. Thus, the marked increase in admissions for substance use services in Maryland may not generalize to carve-outs in other states. Fourth, because of the observational nature of this study, all estimates should be interpreted as associations.

## Conclusions

Access to substance use services is vital for reducing unnecessary morbidity and mortality among persons with SUD. Findings from this cross-sectional study suggest that changes between FFS vs MMC coverage for Medicaid-financed SUD services can result in significant changes in treatment utilization. Carving out services may be associated with increases in SUD treatment utilization but with heterogeneous effects across states and treatment types. The results of this study suggest that additional research is needed to ensure vulnerable populations, such as individuals with SUD, are not adversely affected by changes in payment models for substance use services. State Medicaid programs are uniquely positioned to shape access to evidence-based behavioral health services, but it is vital that coverage—whether administered through managed care arrangements or on an FFS basis—is designed to facilitate rather than inhibit access.

## Notes

Supplement. **eAppendix.** Supplemental Methods
eReferences
**eTable 1.** State Weights in Synthetic Control Models for Maryland **eTable 2.** State Weights in Synthetic Control Models for Nebraska **eTable 3.** Changes in Per Capita Admissions for Substance Use Treatment Associated With Changes in MMC Coverage, Unrestricted Donor Pool
