# Supplementary material for: Association Between Medicaid Managed Care Coverage of Substance Use Services and Treatment Utilization
Source: JAMA Health Forum. 2022 Aug 26;3(8):e222812. doi: 10.1001/jamahealthforum.2022.2812 (PMC9419018; doi:10.1001/jamahealthforum.2022.2812)
Supplement: Supplement. — eAppendix. Supplemental Methods eReferences eTable 1. State Weights in Synthetic Control Models for Maryland eTable 2. State Weights in Synthetic Control Models for Nebraska eTable 3. Changes in Per Capita Admissions for Substance Use Treatment Associated With Changes in MMC Coverage, Unrestricted Donor Pool [file jamahealthforum-e222812-s001.pdf]

Supplemental Online Content

Auty SG, Cole MB, Wallace J. Association between Medicaid managed care coverage of substance use services and treatment utilization. *JAMA Health Forum*. 2022;3(8):e222812. doi:10.1001/jamahealthforum.2022.2812

eAppendix. Supplemental Methods

eReferences

eTable 1. State Weights in Synthetic Control Models for Maryland

eTable 2. State Weights in Synthetic Control Models for Nebraska

eTable 3. Changes in Per Capita Admissions for Substance Use Treatment Associated With Changes in MMC Coverage, Unrestricted Donor Pool

This supplemental material has been provided by the authors to give readers additional information about their work.

© 2022 Auty SG et al. *JAMA Health Forum.*

eAppendix. Supplemental Methods

Study Data and Sample

The study period ran from January 1, 2010 to December 31st, 2019. Substance use treatment admissions were obtained from the Treatment Episode Data Set - Admissions (TEDS-A), collected by the Substance Abuse and Mental Health Services Administration. The TEDS-A collects national data on admissions to substance abuse treatment facilities. In all states, treatment programs receiving any public funds are required to provide data on all admissions. The TEDS-A capture a majority of all admissions to substance use treatment facilities.[1]

Per established synthetic control methods (SCM),[2] we curated the donor pool by excluding states that did not use comprehensive Medicaid managed care (MMC) to deliver Medicaid coverage during the study period (Alabama, Alaska, Arkansas, Connecticut, Idaho, Maine, Montana, North Carolina, North Dakota, Oklahoma, South Dakota, Vermont, and Wyoming), transitioned from Medicaid fee-for-service to MMC during the study period (Iowa) or changed their coverage mechanism for substance use services (Arizona) during the post-period of treated states (i.e. Maryland and Nebraska). The donor pool included California, Colorado Delaware, the District of Columbia, Florida, Hawaii, Illinois, Iowa, Kansas, Kentucky, Louisiana, Massachusetts, Michigan, Minnesota, Mississippi, Missouri, Nevada, New Hampshire, New Jersey, New Mexico, New York, Pennsylvania, Rhode Island, Tennessee, Texas, Utah, Virginia, West Virginia, and Wisconsin. Our final analytic dataset included 310 state-years of data from 30 states and the District of Columbia.

Outcome Variables

We calculated our outcome variables annually as follows:

$$\text{Substance use treatment admissions per 100,000 residents} = \frac{Total\ admissions}{State\ population} \times 100{,}000$$

Unadjusted Comparisons

Unadjusted changes in substance use treatment admissions were identified by comparing the three years preceding the carve-out (Maryland pre-period: 2012, 2013, 2014) or carve-in (Nebraska pre-period: 2014, 2015, 2016) with the following three years. Significance levels and confidence intervals were calculated using two-sided t-tests.

Synthetic Control Comparisons

Synthetic control methods (SCM) create a synthetic version of the treated unit using data from non-treated units in a donor pool. The synthetic control is a weighted average of states in the donor pool, with the weights chosen so that pre-trends in covariates and/or outcomes are as similar as possible between the treated state(s) and synthetic control.[2,3] Weights are selected using a data-driven algorithm. For synthetic control estimation, we used data from the four years prior to the policy change in each state (2010-2014 in Maryland, 2012-2016 in Nebraska).

In this study, we opted to match on pre-trends of the following variables for the entire pre-period: opioid overdose deaths per 100,000 residents, percent of Medicaid beneficiaries enrolled in MMC, substance use treatment facilities per 100,000 residents, earmarked substance use treatment facilities per 100,000 residents, and Medicaid expansion status. We also matched on substance use treatment admissions in the first year of the pre-period (i.e. lagged). Matching on outcomes for the entire pre-period can inflate effect estimates,[4] and lagging outcome data can mitigate this potential bias. This vector of

© 2022 Auty SG et al. *JAMA Health Forum.*

covariates best minimized root mean square prediction error (MSPE) and approximated pre-trends in the outcome between the treated states and resulting synthetic control(s).

We tested alternative matching algorithms by constructing synthetic controls using different covariates, including Medicaid enrollment and state use of MMC "In Lieu of" authority, which allows MMC plans to cover services delivered at institutes of mental disease (IMD) and receive federal matching funds for those enrollees. Synthetic controls constructed with these covariates produced a worse match with the treated states, but did not change the conclusions of results.

To estimate synthetic controls, we followed the approach outlined in Robbins, Saunders, and Kilmer (2021) using the package 'microsynth' for R Statistical Software version 4.1.3.[5] Separate synthetic controls models were estimated for each treated state. Taylor series linearization (TSL) was used to calculate 95% confidence intervals for the effect of MMC coverage changes on treatment admissions in the following two years. We limited the post period to two-years to avoid exposure to other policy changes in these states that might impact SUD treatment admissions and bias estimates. We then conducted a series of permutation tests to determine placebo effect sizes by iteratively reassigning treatment status to each state in the donor pool and re-running synthetic control models, which generates placebo effect sizes.

Robustness Tests

To assess if the impact of carve-outs were driven by changes in admissions among Medicaid enrollees, we re-ran synthetic control models stratified by source of health insurance coverage (uninsured, Medicaid, commercial, and Medicare/other) using states with a low degree of missingness in this variable. Twenty-seven states reported source of insurance coverage for at least 75% of admissions during the study period and were included in these stratified analyses. Admissions covered by Medicaid were calculated as the proportion of admissions per 100,000 Medicaid enrollees, and all other admissions were per 100,000 residents. Synthetic control estimation for this stratification followed the primary approach described in the manuscript. The approach outlined by Catteneo et al. to obtain prediction intervals for synthetic control models was implemented using the R package "scpi."[6] We estimated synthetic control estimation and inference procedures using ordinary least square constraints for synthetic control weights. Models were also re-estimated using the approach outlined by Cattaneo et al. in their empirical example, wherein the authors varied the approach to quantify in and out-of-sample uncertainty.

eReferences

1. Mutter R, Ali MM, Smith K, Strashny A. Factors associated with substance use treatment completion in residential facilities. *Drug Alcohol Depend*. 2015;154:291-295.
2. Abadie A. Using synthetic controls: Feasibility, data requirements, and methodological aspects. *J Econ Lit*. 2019.
3. Athey S, Imbens GW. The state of applied econometrics: Causality and policy evaluation. *J Econ Perspect*. 2017;31(2):3-32.
4. Doudchenko N, Imbens GW. *Balancing, Regression, Difference-in-Differences and Synthetic Control Methods: A Synthesis*. National Bureau of Economic Research; 2016.
5. Robbins MW, Davenport S. microsynth: Synthetic Control Methods for Disaggregated and Micro-Level Data in R. *J Stat Software; Vol 1, Issue 2* . January 2021. https://www.jstatsoft.org/v097/i02.
6. Cattaneo MD, Feng Y, Titiunik R. Prediction intervals for synthetic control methods. *J Am Stat Assoc*. 2021;116(536):1865-1880.

© 2022 Auty SG et al. *JAMA Health Forum.*

**eTable 1.** State Weights in Synthetic Control Models for Maryland				
State	All Admissions	Outpatient Admissions	Detox Admissions	Residential Admissions
California	0%	0%	0%	0%
Delaware	0%	0%	0%	0%
District of Columbia	52%	29%	9%	12%
Florida	0%	0%	7%	0%
Hawaii	0%	0%	18%	16%
Illinois	0%	0%	0%	0%
Indiana	0%	0%	0%	0%
Kansas	20%	0%	0%	0%
Kentucky	0%	0%	0%	1%
Louisiana	0%	0%	0%	71%
Massachusetts	0%	0%	0%	0%
Michigan	0%	0%	0%	0%
Minnesota	0%	0%	23%	0%
Mississippi	0%	0%	0%	0%
Missouri	0%	0%	0%	0%
Nevada	0%	0%	0%	0%
New Hampshire	0%	0%	1%	0%
New Jersey	10%	2%	5%	0%
New Mexico	0%	0%	0%	0%
New York	19%	33%	0%	0%
Ohio	0%	0%	18%	0%
Pennsylvania	0%	0%	0%	0%
Rhode Island	0%	36%	17%	0%
Tennessee	0%	0%	0%	0%
Texas	0%	0%	0%	0%
Utah	0%	0%	0%	0%
Virginia	0%	0%	0%	0%
West Virginia	0%	0%	0%	0%
Wisconsin	0%	0%	2%	0%

© 2022 Auty SG et al. *JAMA Health Forum.*

Etable 2. State Weights in Synthetic Control Models for nebraska

State	All Admissions	Outpatient Admissions	Detox Admissions	Residential Admissions
California	0%	0%	0%	0%
District of Columbia	0%	0%	0%	0%
Delaware	0%	0%	0%	0%
Florida	0%	0%	0%	0%
Hawaii	0%	0%	14%	12%
Illinois	0%	0%	0%	0%
Indiana	0%	0%	0%	0%
Kansas	21%	0%	0%	38%
Kentucky	0%	0%	0%	0%
Louisiana	0%	0%	0%	0%
Massachusetts	0%	0%	0%	0%
Michigan	0%	0%	0%	0%
Minnesota	61%	42%	0%	12%
Missouri	0%	0%	0%	0%
Mississippi	0%	38%	0%	0%
New Hampshire	0%	0%	0%	0%
New Jersey	0%	0%	0%	0%
New Mexico	0%	0%	0%	0%
Nevada	0%	0%	0%	0%
New York	0%	0%	72%	0%
Ohio	0%	0%	0%	0%
Pennsylvania	0%	0%	0%	0%
Rhode Island	0%	0%	0%	0%
Tennessee	0%	0%	0%	0%
Texas	18%	21%	14%	39%
Utah	0%	0%	0%	0%
Virginia	0%	0%	0%	0%
Wisconsin	0%	0%	0%	0%

© 2022 Auty SG et al. *JAMA Health Forum.*

eTable 3. Changes in Per Capita Admissions for Substance Use Treatment Associated With Changes in MMC Coverage, Unrestricted Donor Pool

**Outcome**	**Synthetic Control Estimates[b], (95% CI)**
	**% Change**
Carve-out: Maryland	
All Admissions	+85.6 (31.8, 161.2)***
Outpatient admissions	+158.3 (90.3, 250.7)***
Rehab/residential admissions	-40.4 (-58.0, -15.5)***
Detox admissions	-34.9 (-66.7, 27.3)
Carve-in: Nebraska	
All Admissions	-44.1 (-54.1, -32.0)*
Outpatient admissions	+36.7 (-12.6, 113.9)
Rehab/residential admissions	+6.5 (-20.3, 42.3)
Detox admissions	-31.9 (-53.2, -0.9)***

Abbreviations: MMC – Medicaid managed care; SD – standard deviation; CI – confidence interval.
[a] Mean outcome during the 5-years immediately preceding and 2-years following changes in MMC coverage of substance use services.
[b] Synthetic control estimates for the % change in outcomes during the two-years post coverage change. Significance is based on permutation testing, and may not align with confidence intervals obtained from Taylor Series Linearization.
[c] Per 100,000 state residents

© 2022 Auty SG et al. *JAMA Health Forum.*
